# Supplementary material for: The spreading of SARS-CoV-2: Interage contacts and networks degree distribution
Source: PLoS One. 2021 Aug 25;16(8):e0256036. doi: 10.1371/journal.pone.0256036 (PMC8386875; doi:10.1371/journal.pone.0256036)
Supplement: S1 Appendix — (DOCX) [file pone.0256036.s001.docx]

# S1 Appendix. Polymod data analyses


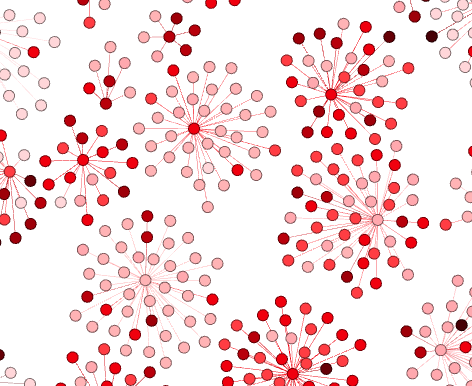


Figure S1: Sample illustrative of the reconstructed, ego-centered network of Italy based on Polymod data.

Colors represent ten-year age bands. Respondents to the survey are at the center of each star, and linked to the contacts they declared.


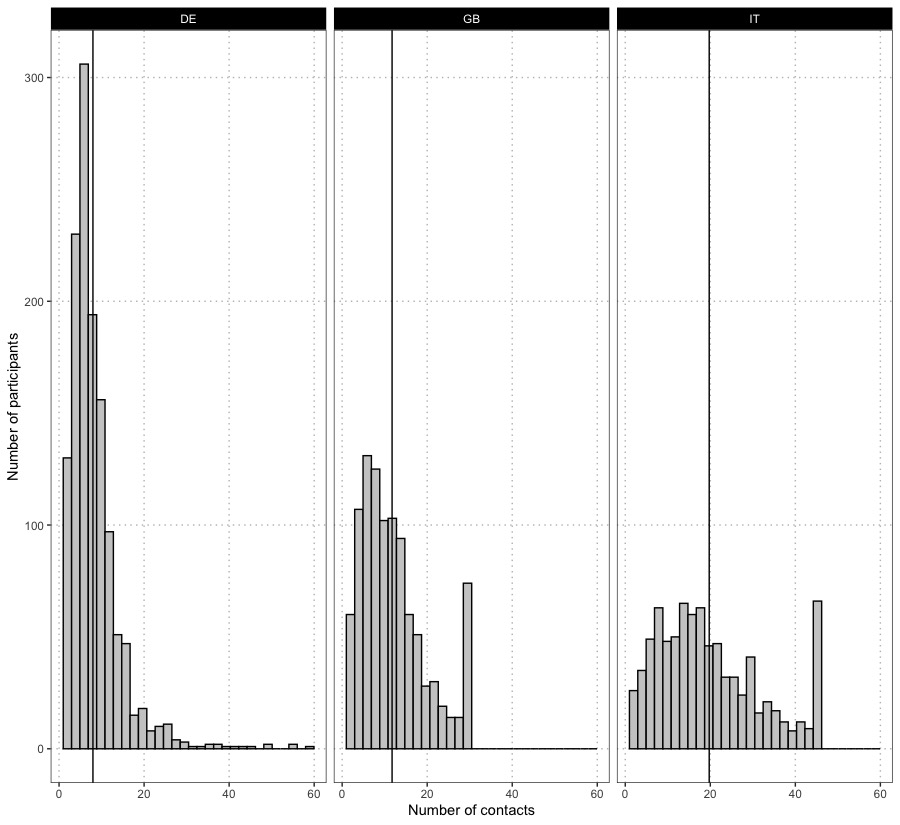


Figure S2: Distribution of number of contacts in Polymod data.

Vertical black lines display country mean.


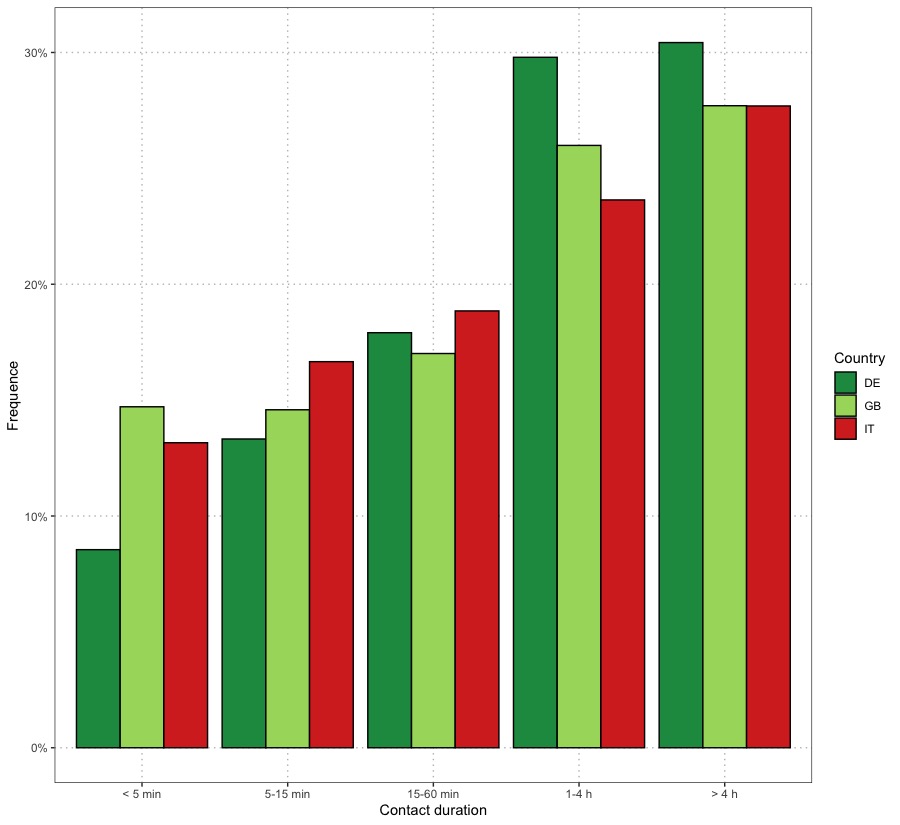


Figure S3: Distribution of contact duration across countries based on Polymod data.

There are a few caveats in the Polymod data. Although the survey was designed to make data comparable across countries, different maximum number of reported contacts were allowed (Germany=73; Great Britain=30; Italy=45). It is not rare in Italy and Great Britain for participants to have reported the maximum, which explain the peak at the extreme right of the distributions Fig S2. This may mean that the average number of contacts in these countries is understated when compared to Germany. We preserve the data with different top coding thresholds, because participants in Germany were asked not to report more than ten contacts occurring in the workplace, which limit was not imposed in Italy and Great Britain. Although this limits the comparability of our data, the workplace limitation in Germany may be partly compensated by the overall contacts limits in the other countries. Moreover, we believe it extremely unlikely that this difference in the data collection process is the only cause of the very large difference we observe between countries.

Importantly, the correlation between the number of daily contacts reported by participants and the duration of these contacts is both very weak and negative (Pearson correlation coefficient= −0.031 pooling the three countries together), which is in line with the results reported by Manzo and van de Rijt (2020) for France. This implies that individuals with high numbers of daily contacts do not have significantly shorter contacts on average. Similarly, we find that having more daily contacts reduces the probability that each of these contacts is physical only slightly. We ran a simple linear regression and found that for a 1% increase in the number of a participant’s daily contacts, the probability of the contact being physical reduces by 0.00077459. Though statistically significant, we interpret this effect as substantively negligible.

Another concern is that countries reporting a lower average number of contacts could somehow compensate if these contacts were longer. Fig S3 shows that this is partially the case: Germans have a lower proportion of very short contacts, and a higher proportion of longer contacts. Although we do not know how long each contact lasted, only the interval it fell into, we need to make assumptions in order to estimate the average contact duration in each country. For shorter intervals we took the value mid-way between the upper and lower bounds, and for the “*more than four hours*” interval we arbitrarily assumed contacts lasted on average six hours. Doing this, we found that the average German contact lasted 162 minutes, with 147 minutes for Great Britain and 144 minutes for Italy. Put differently, on average German contacts and British contacts lasted (respectively) 12.5% and about 2.1% longer than Italian contacts. This could partially compensate the average fewer daily contacts reported by German participants. We took these differences into account by increasing the probability of dyadic contagion (see “Diffusion of the virus: agent-based SEIR model”).
