# Supplementary material for: The spreading of SARS-CoV-2: Interage contacts and networks degree distribution
Source: PLoS One. 2021 Aug 25;16(8):e0256036. doi: 10.1371/journal.pone.0256036 (PMC8386875; doi:10.1371/journal.pone.0256036)
Supplement: S2 Appendix — (DOCX) [file pone.0256036.s002.docx]

# S2 Appendix. Description of the algorithm to reduce total age-distance D

The networks we generate need to match two empirical characteristics simultaneously: first, nodes must have a number of links precisely equal to their desired degree, with no self-links and with a maximum of one link between each pair of nodes; second, the total age-distance must be minimized. Given these two criteria, we decided to decompose the problem into two steps: first building a network that respects the degree distribution, then progressively reducing the total age-distance by replacing blocks of links.

The difficulty in reconstructing a network that respects the age mix comes from the fact that when one node desires to create a link with another, that node should reciprocate because links are undirected. Of course, if the dataset at our disposal covered the daily contacts of a country’s whole population, and not just a sample of that population, every contact would be listed twice—once per individual in the dyadic interaction—and there would be an optimal distribution with total age-distance D=0. In practice, only having a sample leads to mismatches between the declarations of the participants. It is not rare that respondents of age *a_1_* declared contacts with individuals of age *a_2_*, but that no participant of age *a_2_* reported a contact with someone of age *a_1_*.

Several factors reinforce these sorts of “reciprocity” issues. Firstly, participants reported an estimation of the age of their contacts. This probably introduced some mistakes. Secondly, we have modified the population age structure by imposing another country’s structure, or the same country’s age structure but in 2020, not when the survey was conducted.

Our first step, matching the empirical degree distribution, is described in the text of this paper. Our second step was to progressively replace ties, to minimize age-distance. For this we proceeded iteratively:

1. Select 1,500 links with age-distance *d* greater than the average age-distance (D/L) in the population of links. If there are less than 1,500 links satisfying this condition, then select only the links satisfying the condition.
2. Select each of these links in a random order and delete it—but only if neither of the nodes the link connects has already had a link deleted in this iteration. In other words, no node can have more than one link deleted in an iteration. We call the links deleted “missing” links. All the remaining links are considered “fixed” for the rest of that iteration.
3. Nodes are considered as “available” if they have a missing link, and if they have less than nine “temporary” links (see 3.a.i.). Two nodes connected by a fixed link may not gain a temporary link between them.
   1. While available nodes remain, carry out steps (i) to (vi), at maximum four times:
      1. select each node with a missing link in a random order, and give it “temporary” links to the four available nodes with the smallest age-distance (see equation 2 and subsequent explanations).
      2. remove any node that has both a missing link and more than nine temporary links from the pool of available nodes, but allow it to initiate four temporary links if it has not initiated any yet.
      3. once all such nodes have at least four temporary links, the node with the lowest number of temporary links selects from her temporary neighbors the one with the lowest number of temporary links: this link becomes fixed for the remainder of this iteration; both nodes delete all their remaining temporary links.
      4. if (iii) results in another node being left with only one temporary link, make that link fixed, both that node and its linked neighbor delete all remaining temporary links to their neighbor, and so on.
      5. repeat (iii) and (iv) until no temporary links remain.
   2. If a group of nodes with a missing link remains after running 3.a four times, then randomly create fixed links among them (one per node). If they are already connected through a fixed link, then delete another fixed link (chosen at random from the rest of the population). The nodes formerly connected by the deleted link are now available to link with any nodes that still retain a temporary link after 3.a. We make sure to always respect the condition that no double links exist between each pair of nodes.
   3. Repeat 3.b. until no node with a missing link remain.

Around 100 iterations of the algorithm (from 1. to 3.), the total age-distance D stopped reducing and remained constant. We therefore considered 100 as the threshold for convergence.

| 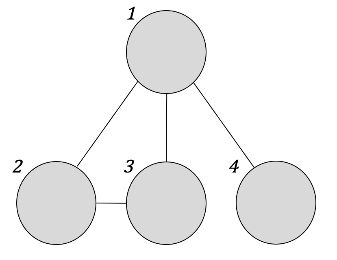 | 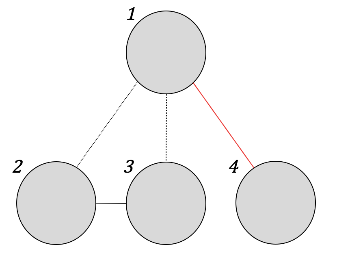 | 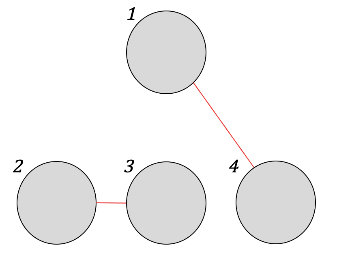 |
| --- | --- | --- |

Figure S4: Illustration of the procedure for deleting temporary links.

To simplify, existing fixed links are not represented.

Node 4 has just one temporary link remaining. This is made fixed (red). Its neighbor (node 1) deletes its other temporary links, to Nodes 2 and 3. These are left with one temporary link, so this becomes fixed. If this temporary link had not existed between them, they would have become isolated until step 3.b.
