# Supplementary material for: The spreading of SARS-CoV-2: Interage contacts and networks degree distribution
Source: PLoS One. 2021 Aug 25;16(8):e0256036. doi: 10.1371/journal.pone.0256036 (PMC8386875; doi:10.1371/journal.pone.0256036)
Supplement: S3 Appendix — (DOCX) [file pone.0256036.s003.docx]

# S3 Appendix. Varying population age structure

In our main analyses, we fixed the population age distribution using UN data for Italy, and analyzed the effect of age mix and degree distribution by alternatively calibrating each of those factors on the Italian data. However, the effect of age mix and degree distribution may depend on the underlying age structure. This may be particularly salient with respect to the age mix if, for instance, members of one age group want to create links with a different age group that represents a smaller proportion of the population. In this appendix, we tackle this question by presenting additional results in which, in addition to age mix and degree distribution, we also vary the population age distribution. We tested 27 combinations: 3 population age distributions * 3 degree distributions * 3 age mixes. In line with our main analyses we simulated 50 networks for each of these 27 combinations, and 50 complete diffusion processes for each of these networks. This gave us 27*50*50=67,500 data points. Fig S5_1 replicates Fig 4 for all nine underlying combinations of population age distribution and degree distribution. Fig S5_2 replicates Fig 5 for all nine underlying combinations of population age distribution and age mix. The differences from our baseline case are negligible, making us confident that our conclusions do not depend on the underlying degree distribution or population age distribution.

| Population Great Britain—Degree distribution Italy |
| --- |
| 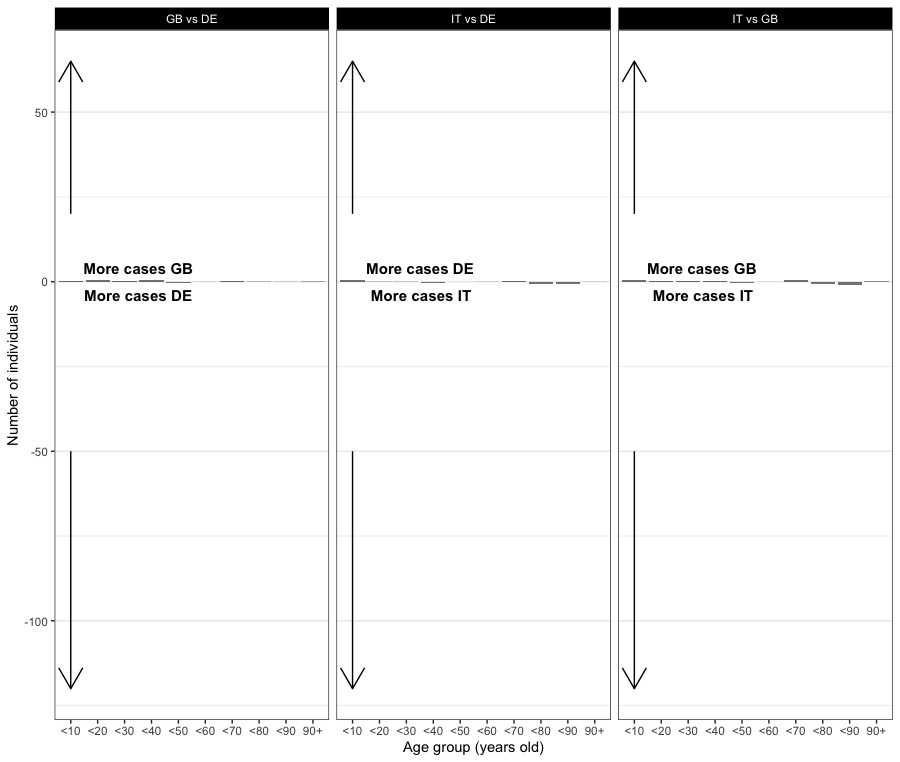 |
| Population Great Britain—Degree distribution Great Britain |
| 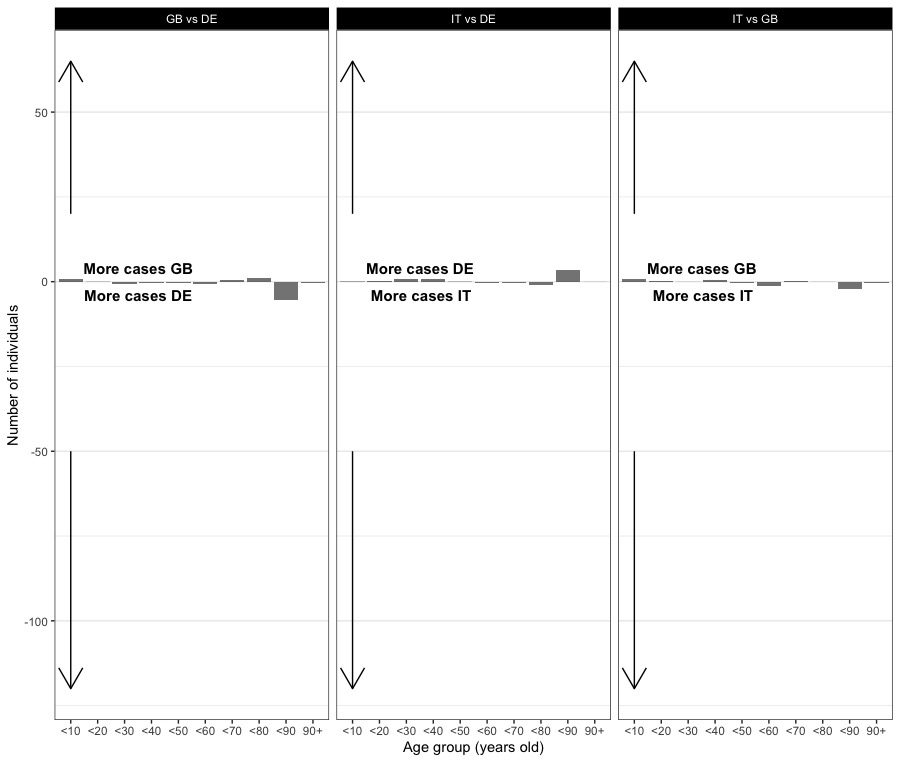 |
| Population Great Britain—Degree distribution Germany |
| 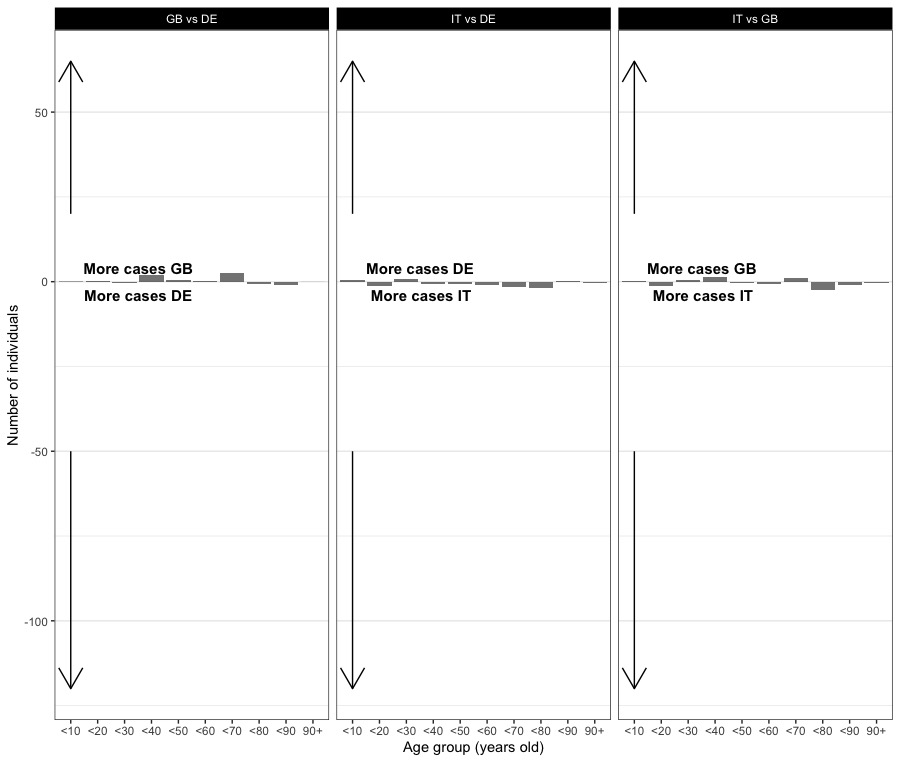 |
| Population Italy—Degree distribution Italy |
| 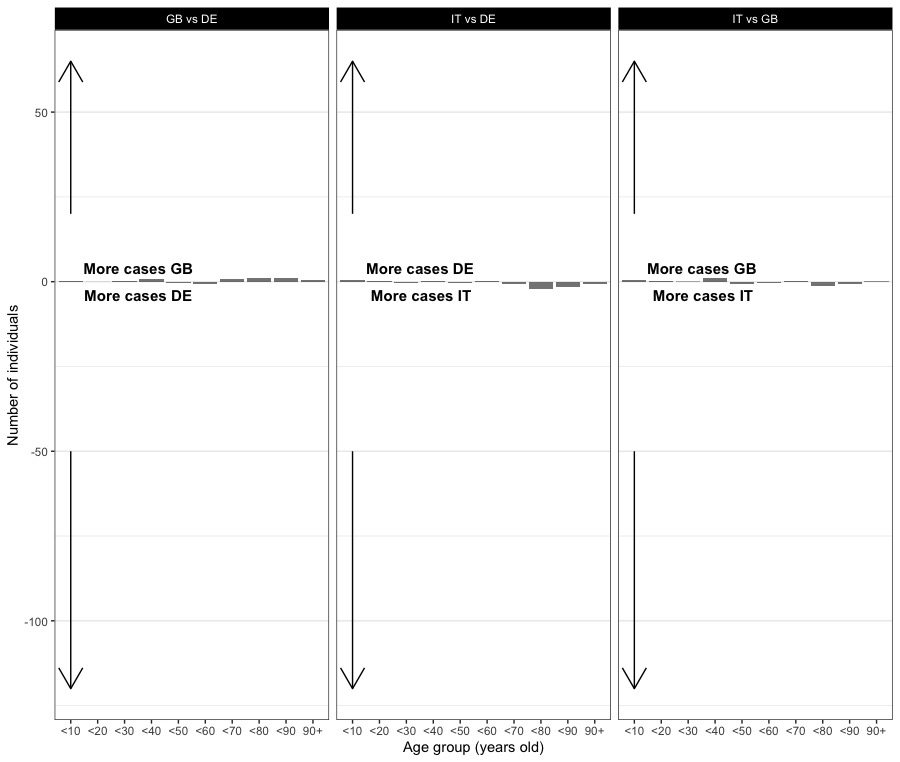 |
| Population Italy—Degree distribution Great Britain |
| 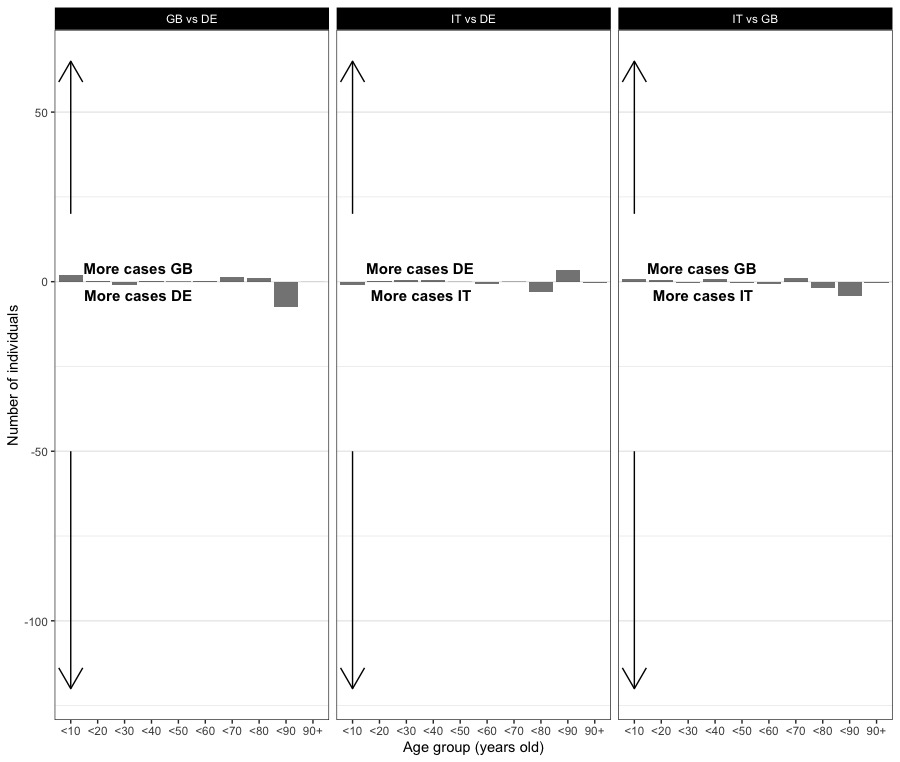 |
| Population Italy—Degree distribution Germany |
| 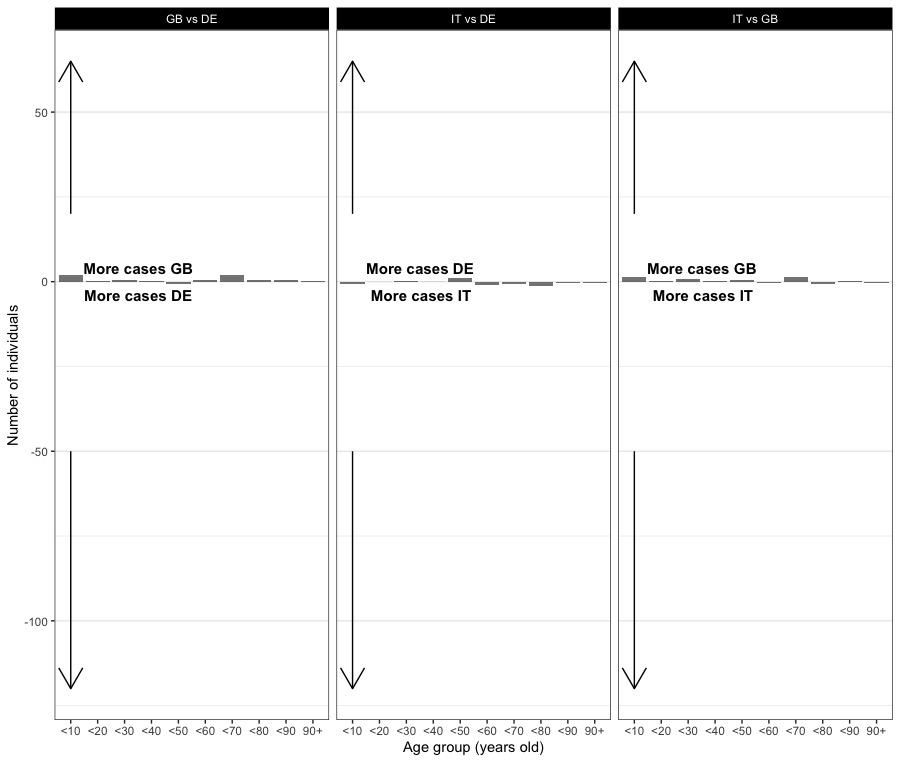 |
| Population Germany—Degree distribution Italy |
| 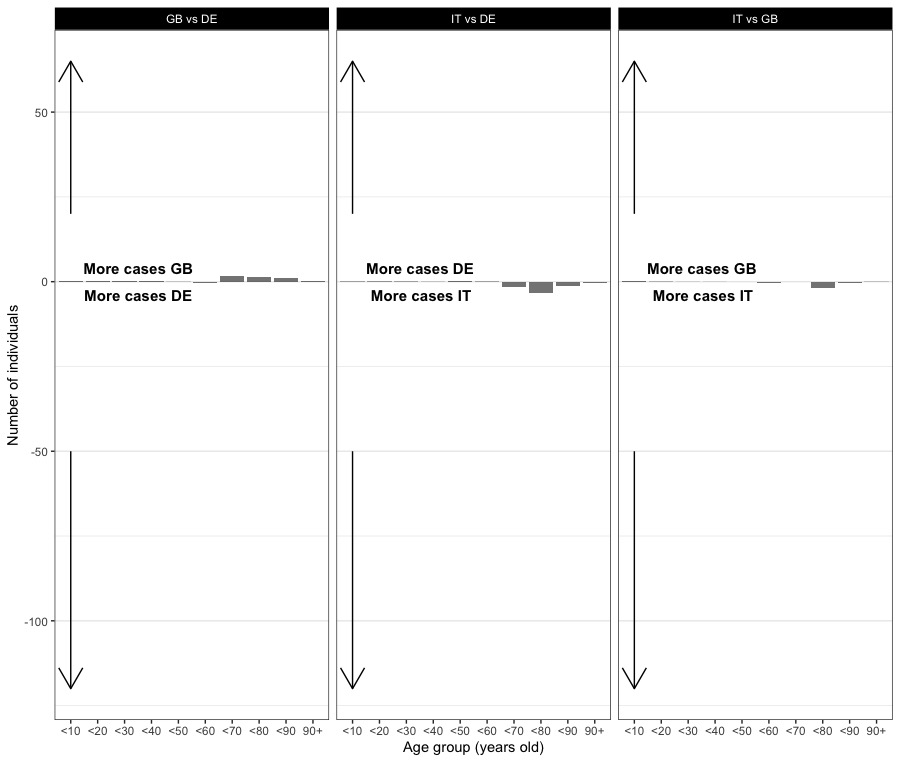 |
| Population Germany—Degree distribution Great Britain |
| 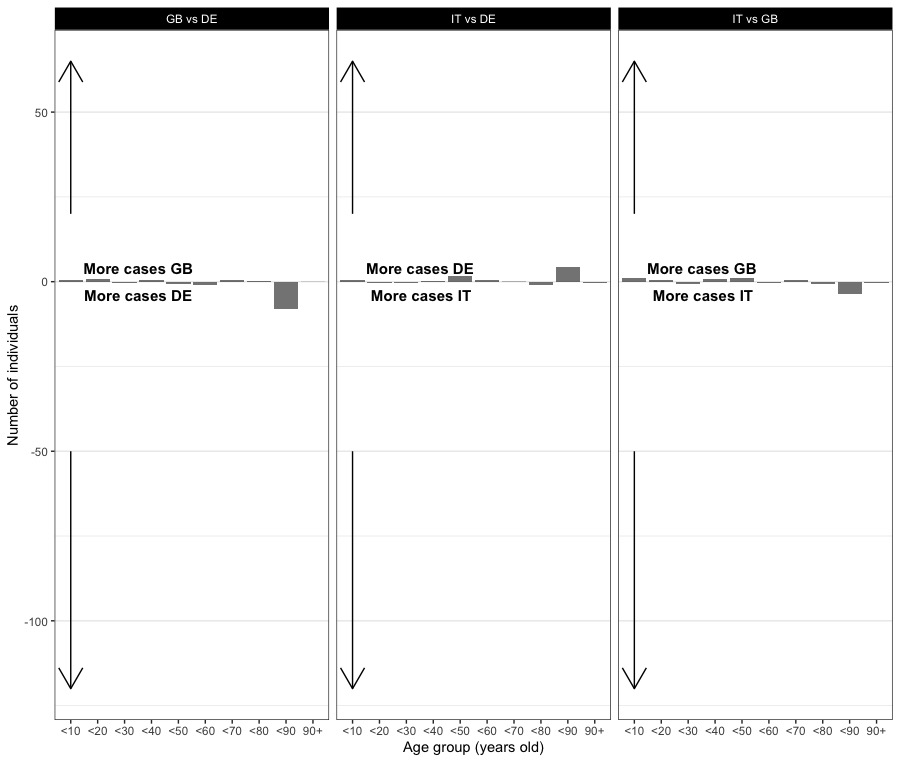 |
| Population Germany—Degree distribution Germany |
| 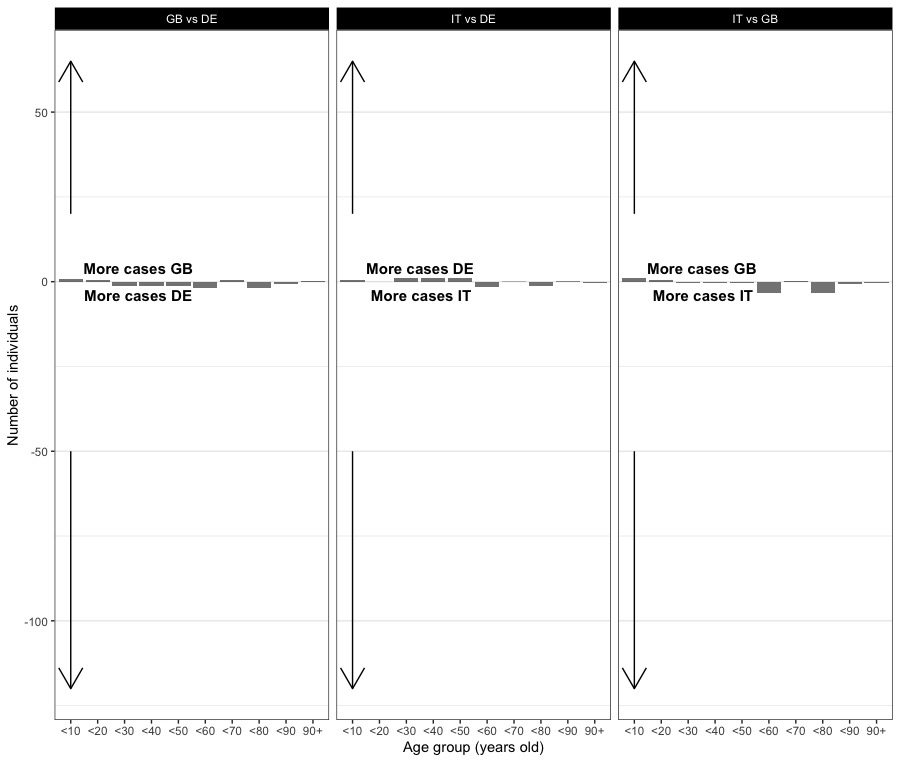 |
| Figure S5_1: Effects of age-mix on diffusion for p=0.05.  Differences in the average number of infected individuals across 10-year age groups at the end of the simulation runs, attributable to age-mix differences for the nine combinations of underlying population age distribution and degree distributions. Left panel compares Great Britain with Germany; central panel Germany with Italy; right panel Great Britain with Italy. The x-axis represents equality: both networks have the same number of infected individuals. |

| Population Great Britain—Age mix Italy |
| --- |
| 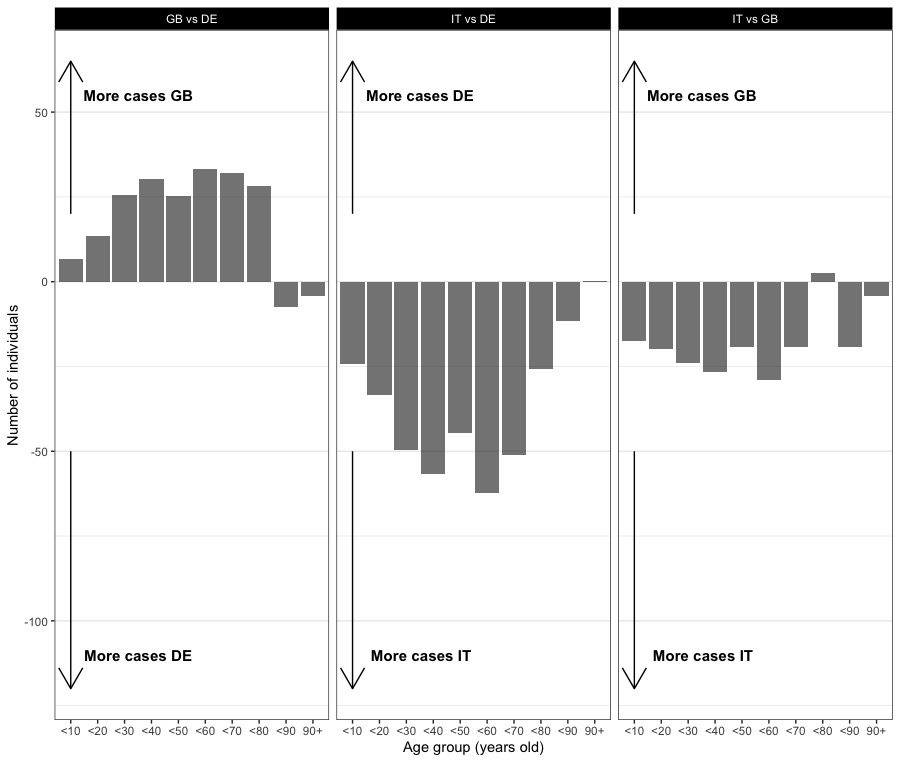 |
| Population Great Britain—Age mix Great Britain |
| 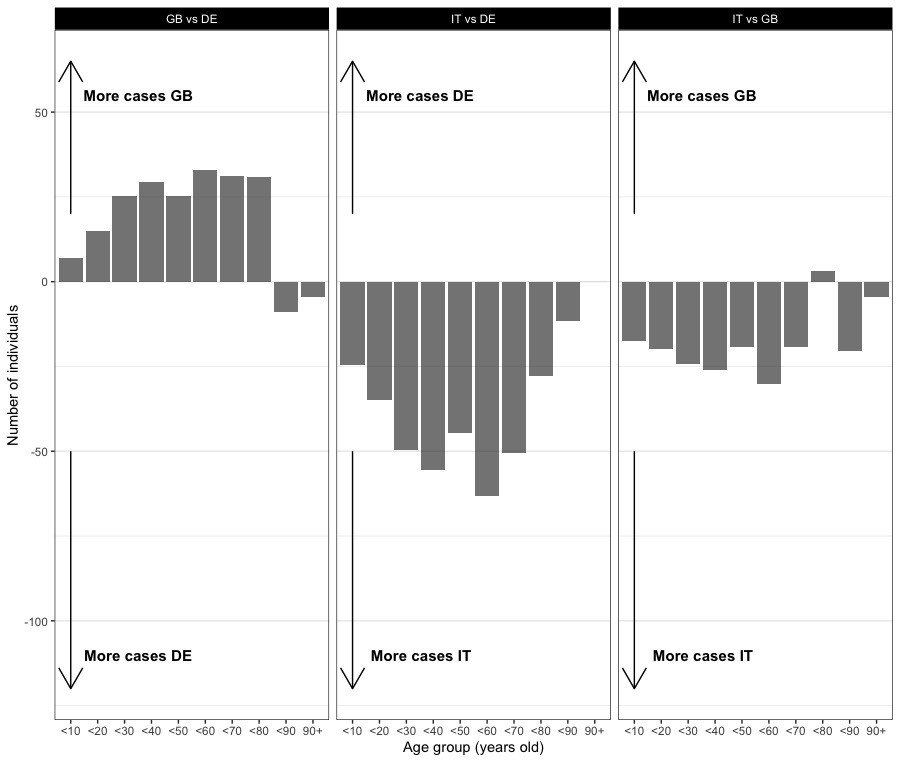 |
| Population Great Britain—Age mix Germany |
| 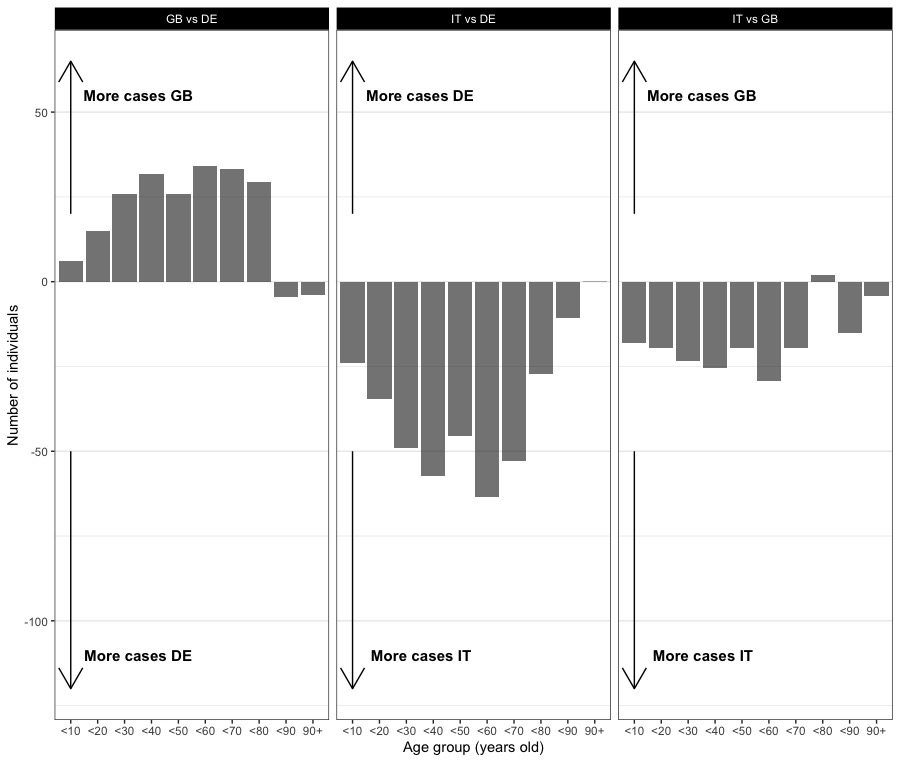 |
| Population Italy—Age mix Italy |
| 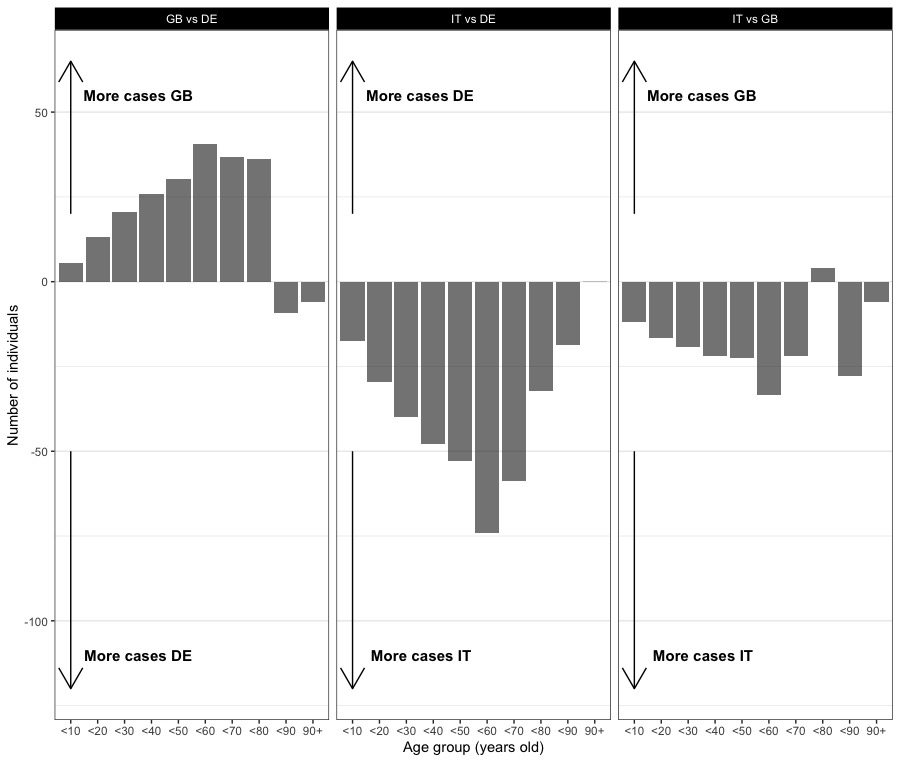 |
| Population Italy—Age mix Great Britain |
| 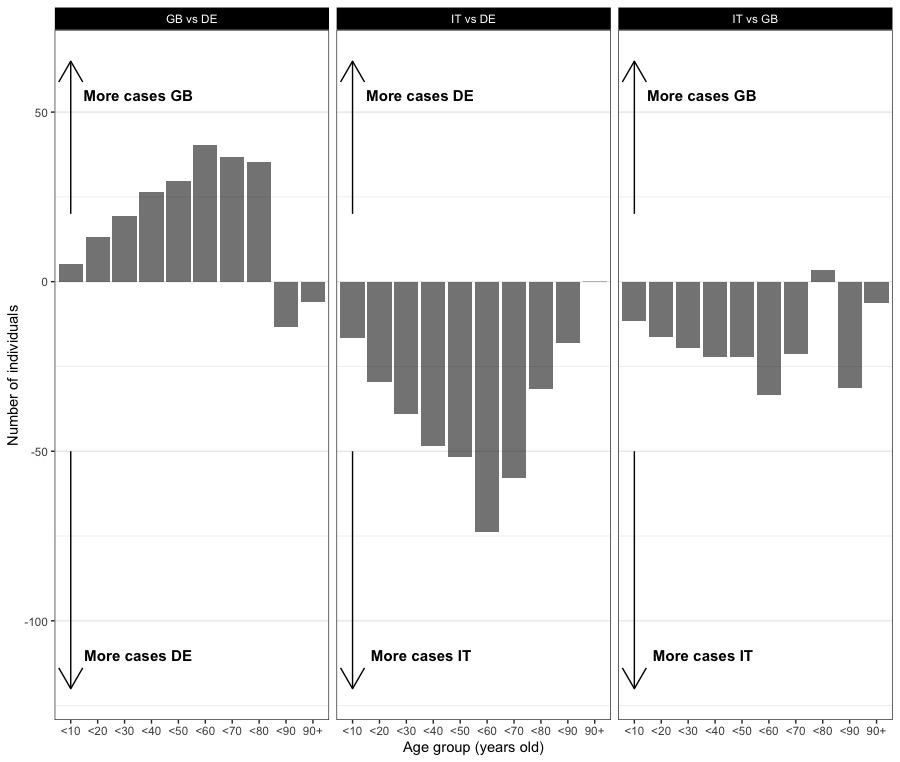 |
| Population Italy—Age mix Germany |
| 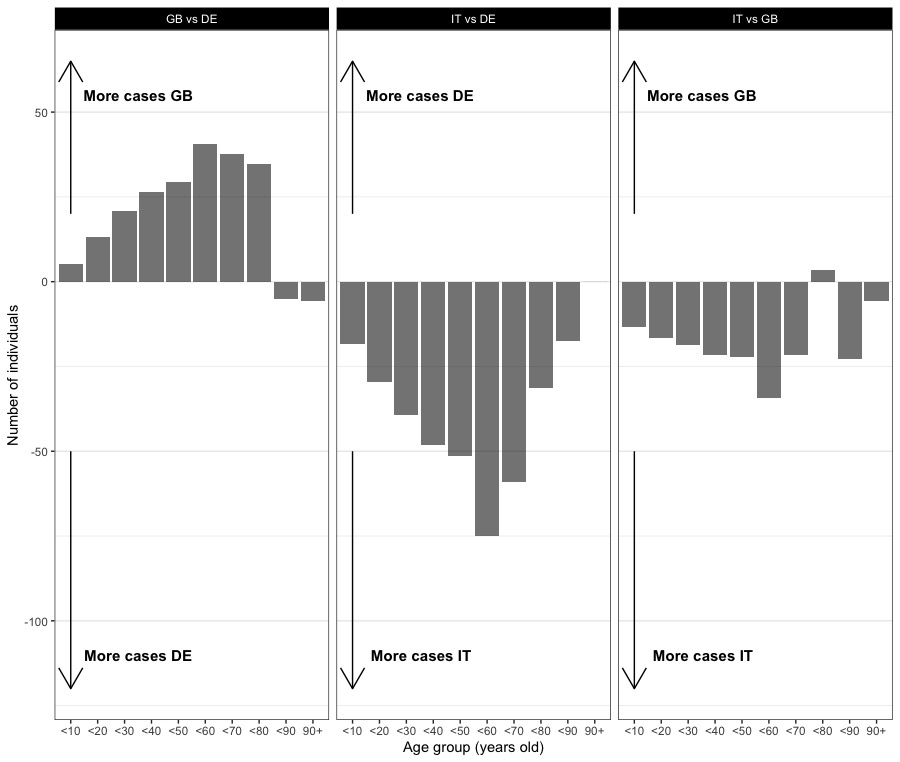 |
| Population Germany—Age mix Italy |
| 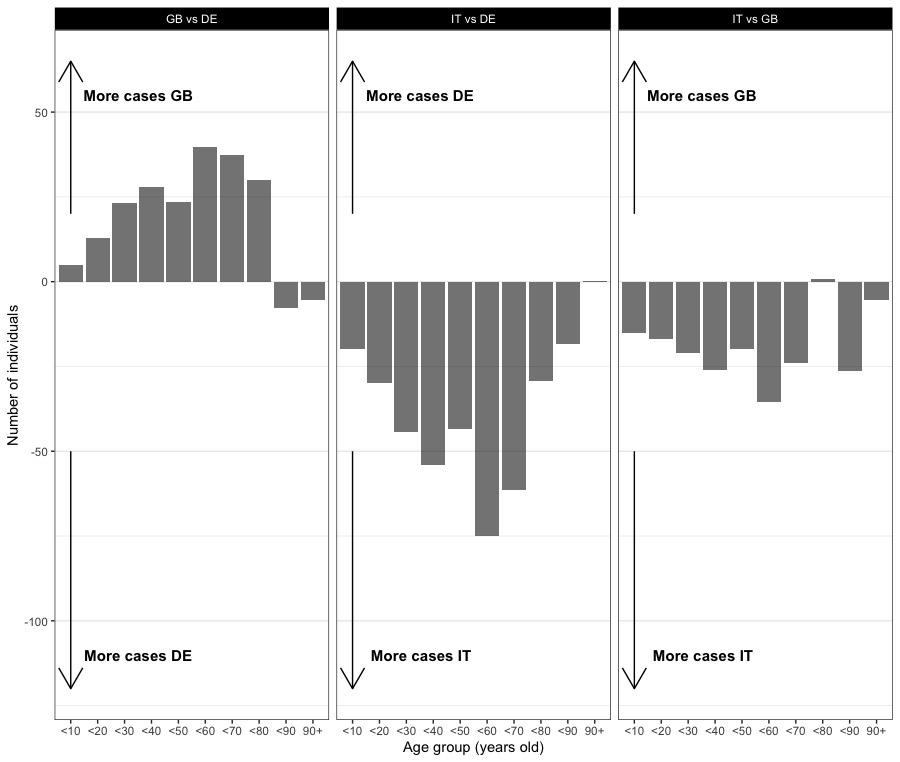 |
| Population Germany—Age mix Great Britain |
| 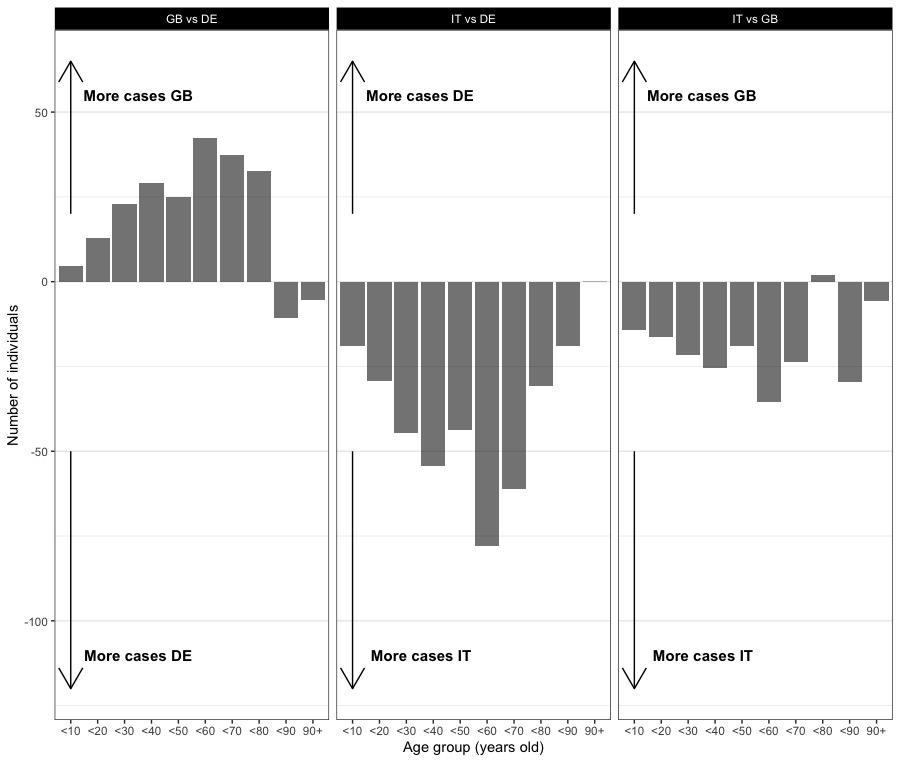 |
| Population Germany—Age mix Germany |
| 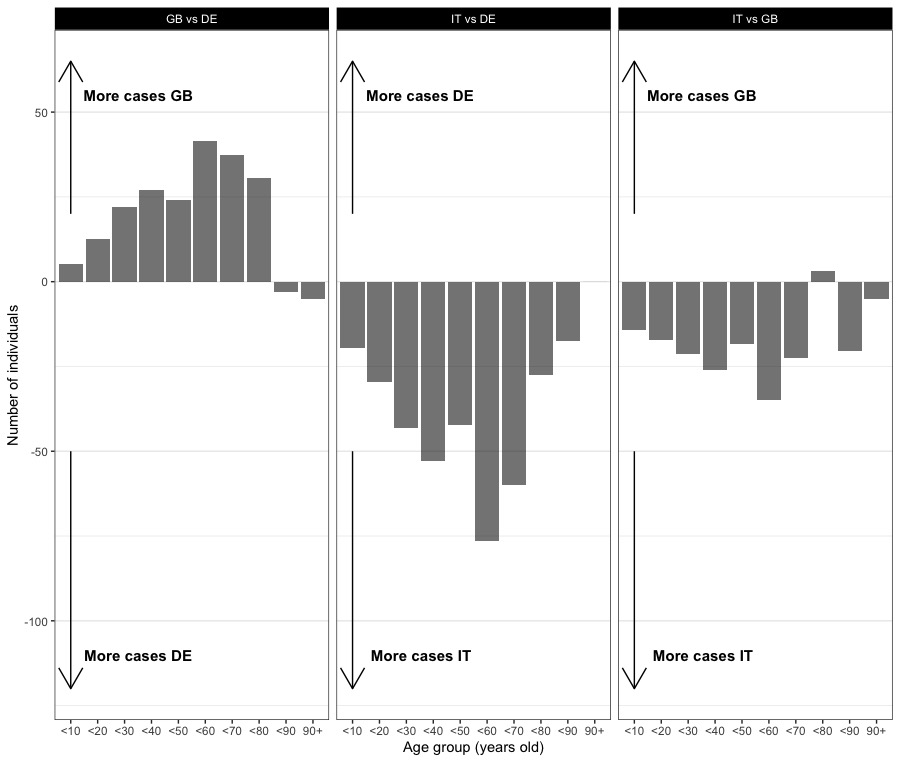 |
| Figure S5_2: Effects of degree distribution on diffusion for p=0.05.  Differences in the average number of infected individuals across 10-year age groups at the end of the simulation runs, attributable to differences in degree distribution for the nine combinations of underlying population age distribution and age mix. Left panel compares Great Britain with Germany; central panel Germany with Italy; right panel Great Britain with Italy. The x-axis represents equality: both networks have the same number of infected individuals. |
