# Supplementary material for: The spreading of SARS-CoV-2: Interage contacts and networks degree distribution
Source: PLoS One. 2021 Aug 25;16(8):e0256036. doi: 10.1371/journal.pone.0256036 (PMC8386875; doi:10.1371/journal.pone.0256036)
Supplement: S4 Appendix — (DOCX) [file pone.0256036.s004.docx]

# S4 Appendix. Generation of networks with tunable level of clustering and age assortativity

To tune the level of clustering, in step 4 of the network generation algorithm, before an individual is removed from the pool of available nodes, we create links between each of her available contacts with a probability *p_clust_*. The algorithm sometimes fails to converge for some degree distributions and some values of *p_clust_*: a few nodes end up with one link fewer than the degree wanted. In such cases, we delete the smallest number of existing links that allows all nodes with one missing link to create the number of links desired.

We cannot run step 5 of the algorithm (described in S2), which aims at matching the age mix of the empirical data, because this would undo the clustering we have just created. To preserve a level age assortativity as close as possible to the “empirical” networks, in step 4, every time a node selects another available node to link with, it chooses the available node closest to itself in age with a probability *p_assort_*. We set *p_assort_* to generate network age-assortativity of 0.44 for Germany and 0.40 for Italy. We make sure that the clustering and age-assortativity generating processes are independent from each other. For this, when node *i* select the available node *j* that is the closest in terms of age with probability *p_assort_*, we make sure that *i* and *j* do not already have a friend in common.
