# Supplementary material for: The spreading of SARS-CoV-2: Interage contacts and networks degree distribution
Source: PLoS One. 2021 Aug 25;16(8):e0256036. doi: 10.1371/journal.pone.0256036 (PMC8386875; doi:10.1371/journal.pone.0256036)
Supplement: S5 Appendix — (DOCX) [file pone.0256036.s005.docx]

# S5 Appendix. Further examination of the roles of age assortativity and clustering

In this appendix, we analyze more systematically the separated and combined effects of age assortativity and clustering on the total number of cases in our populations. In our main results, we found that the empirical differences in age assortativity between the countries did not lead to a major reduction in the number of cases. In this appendix we analyze whether, and if so, to what extent, interage contacts play a role in accelerating/hampering the propagation of the virus. To simplify, we take the Italian population age structure and compare the Italian degree distribution to the German one, then cover the entire range of the parameter space with: $0\leq p_{assort}\leq1$ and $0\leq p_{clust}\leq1$. Figs S6 and S7 respectively report the total proportion of the population that is infected, and the proportion of the elderly population that is infected, with the Italian and the German degree distributions.


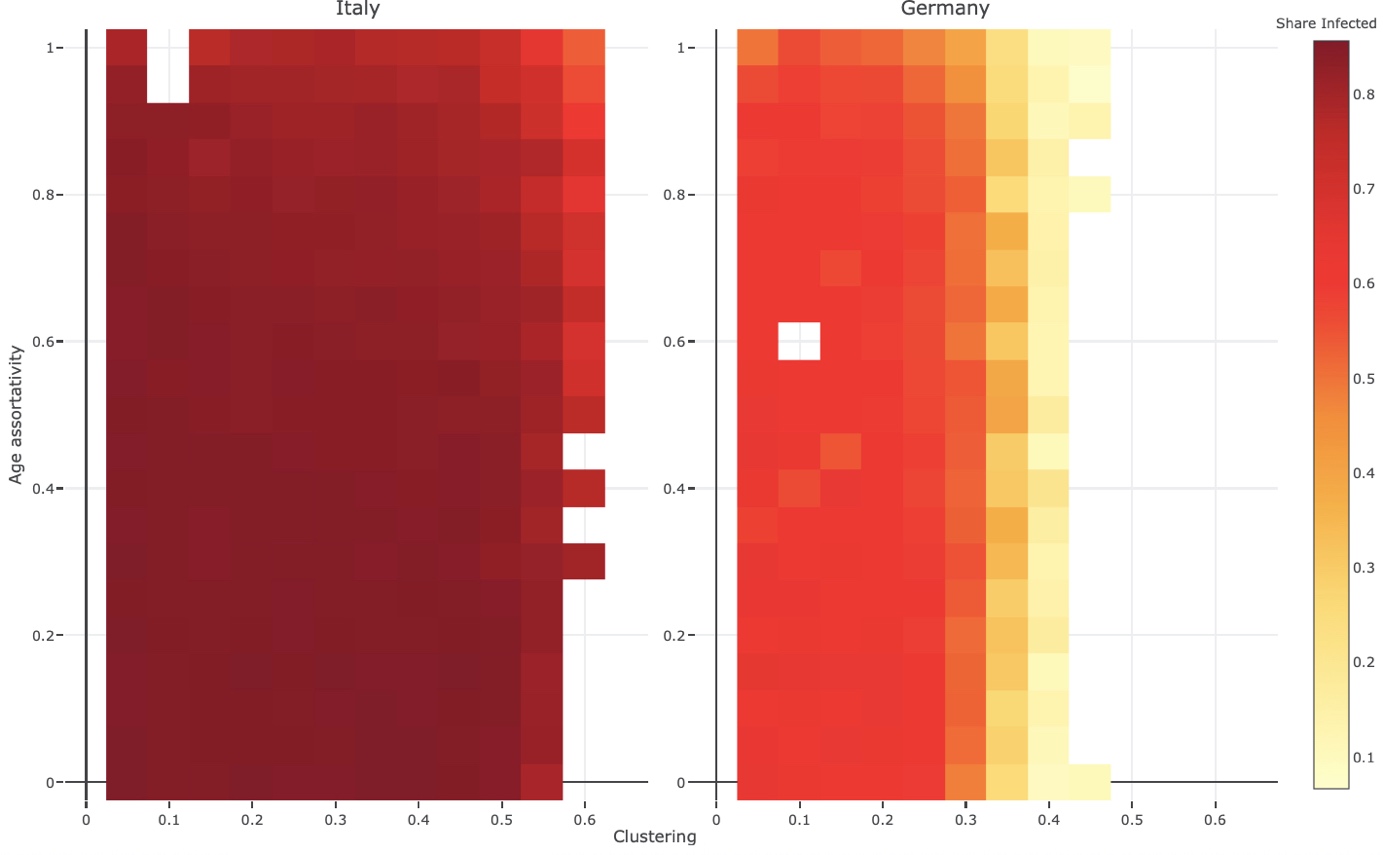


Figure S6: Effects of clustering and age assortativity on the proportion of individuals infected.

Total proportion of cases in networks calibrated on the Italian degree distribution (left panel) and the German degree distribution (right panel). Dyadic contagion probability p=0.05. Italian population age structure. The networks were generated by tuning $0\leq p_{assort}\leq1$ and $0\leq p_{clust}\leq1$ which were incremented by 0.025, i.e., 41 values each. For each network, we simulated the diffusion of the virus five times. In total, each panel is composed of 8,410 data points. We then took the mean of all data points falling within intervals of length 0.05, for clustering coefficient and age assortativity.


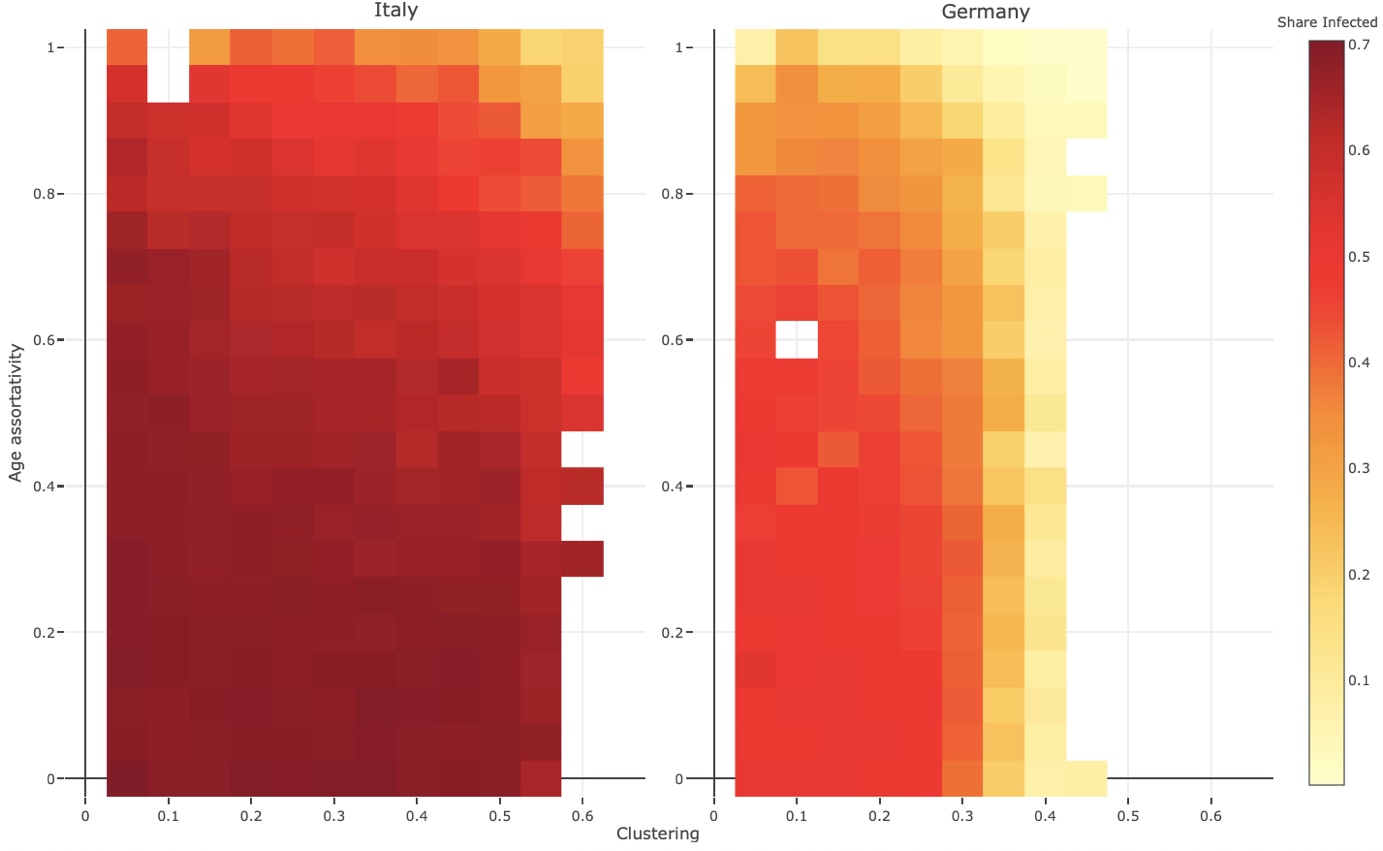


Figure S7: Effects of clustering and age assortativity on the proportion of elderly infected.

Proportion of cases among the elderly in networks calibrated on the Italian degree distribution (left panel) and the German degree distribution (right panel). Dyadic contagion probability p=0.05. Italian population age structure. See Fig S6 for details of figure’s construction.

Several results emerge from this exploration of the parameter space. Firstly, we see that the results for the two countries remain sharply different. Owing to Italy’s degree distribution, the virus spreads more easily in that country than in Germany. The figures indicate, roughly, that maximum values of clustering and age assortativity in Italy would reduce the number of cases there to German levels, only when clustering and age assortativity in Germany are null (i.e., the top right-hand areas of the graphs for Italy are comparable colours to the bottom left-hand areas of the graphs for Germany).

Secondly, we see that clustering can reduce the number of cases, both among the elderly and among the total population, more than age assortativity. For a fixed level of clustering, passing from age assortativity=0 to age assortativity=1 reduces the proportion of cases by about 10 percentage points (more among the elderly). Although this might seem a large reduction in percentage terms, in absolute terms the greatest empirical cross-country difference we found was 0.07 (between Italy and Great Britain). This explains why we found no effect of age assortativity in the main analysis. In other words, empirical country differences in terms of clustering are too low to generate significant country differences in the spread of the virus.

Thirdly, clustering and age assortativity reduce the spread of the virus more across a network calibrated on the German degree distribution than across one calibrated on the Italian degree distribution. Block et al. [16] proposed strategies to flatten the diffusion curve that amount to playing on homophily (in our case age assortativity) and clustering. Their analyses rely on ideal–typical networks with symmetrical degree distribution. Our model points to the crucial role of the empirical degree distribution. The same strategies would likely lead to different results in countries with distributions like Italy and Germany. Fig S8 analyzes the underlying reasons and displays the average shortest path length in the networks. We see that only extreme levels of clustering and age assortativity make a difference in Italy, but that clustering and, to a lower extent, age assortativity significantly reduce the average shortest path length in Germany.


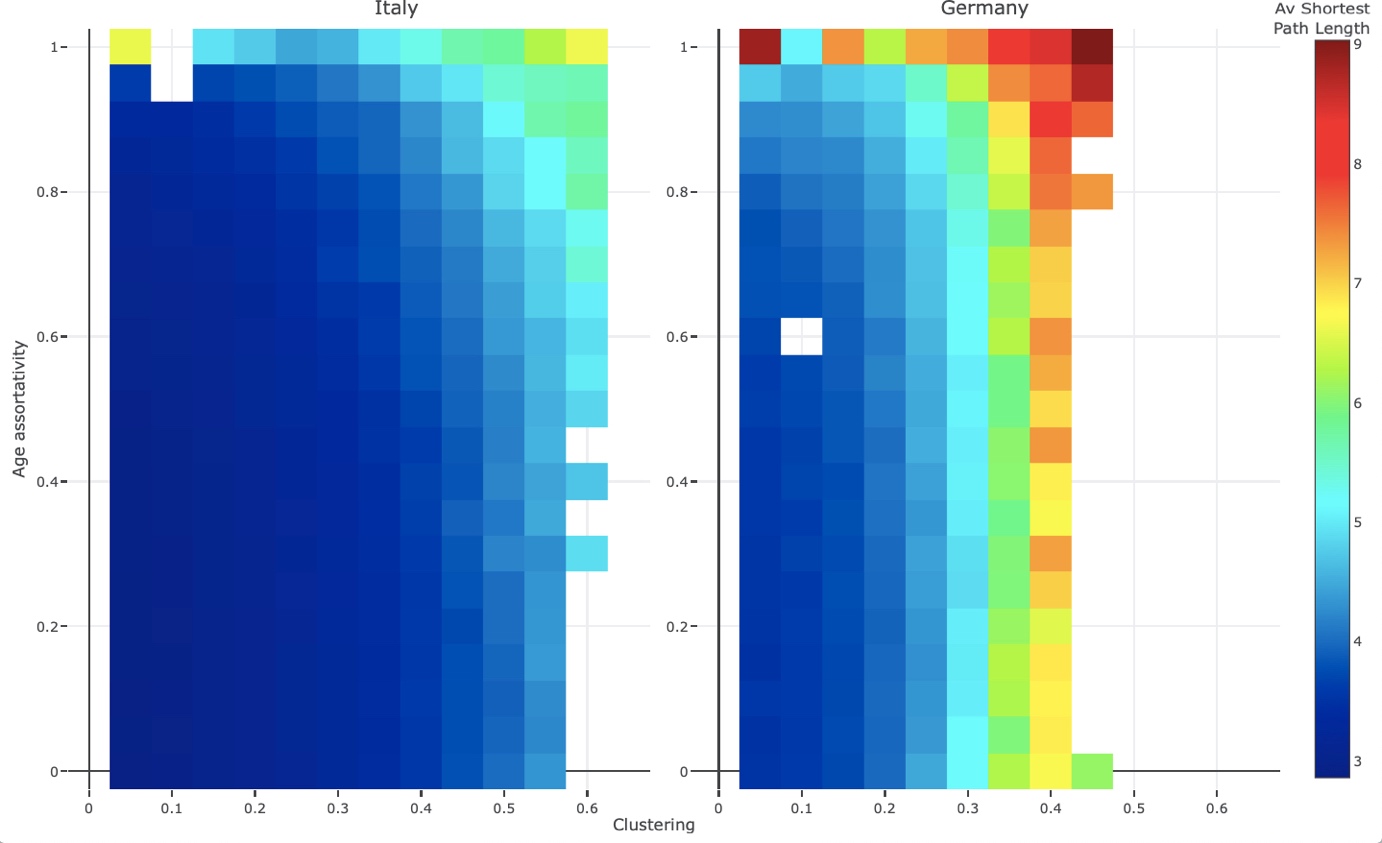


Figure S8: Effects in Italy and Germany of clustering and age assortativity on the average shortest path length.

Average shortest path length in networks calibrated on the Italian degree distribution (left panel) and the German degree distribution (right panel). Italian population age structure. See Fig S6 for details of figure’s construction.

We have mainly been interested in the percentage of cases among the population in general and among the elderly in particular. However, the debate about the ICH—namely the idea that intergenerational differences in age mixing could affect the diffusion of the virus—has also discussed the effect of age mixing on the speed of contagion. Fig S9 analyzes the timing of the contagion peak, that is, the day on which the highest number of individuals were first infected. Although less neat, the patterns are similar to those for the proportion of infected individuals. Strong age assortativity seems to hamper the spread of the disease. However, again, it should be kept in mind that the empirical differences in age assortativity between countries are so small that, on their own, they are unlikely to make significant differences to the timing of the contagion peak.

It is worth recalling that our artificial worlds only consider three factors potentially impacting on the virus diffusion processes: degree distribution, age mixing, and population age structure. How each of these properties may interact with other factors in the real world could increase or decrease their respective importance. Further work, progressively incorporating other relevant factors, is needed to understand whether those factors could interact with age-mixing differences and thereby increase the effects of age mixing on the diffusion of the virus across a network.


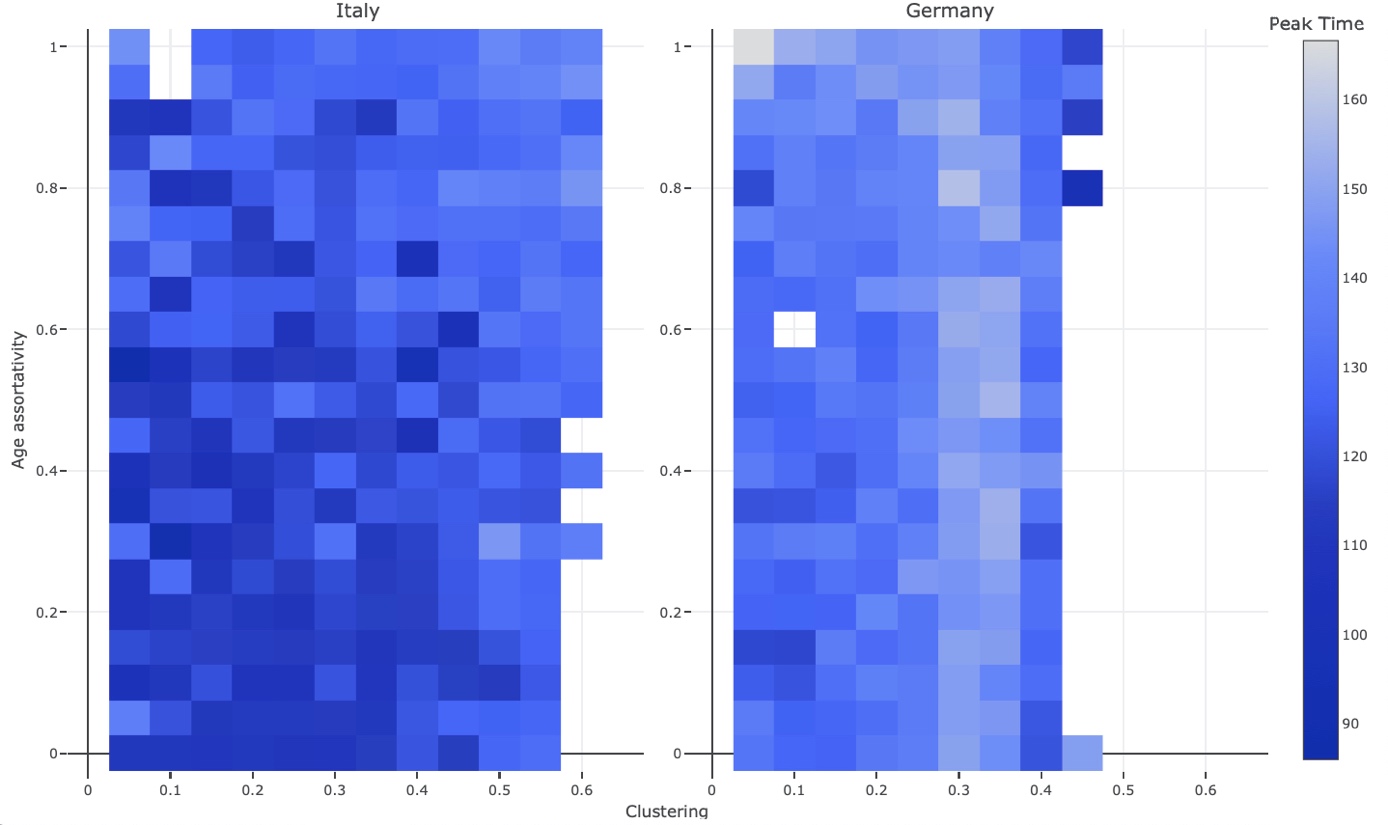


Figure S9: Effects in Italy and Germany of clustering and age assortativity on the timing of the diffusion peak.

Iteration at which the diffusion peak occurs in networks calibrated on the Italian degree distribution (left panel) and the German degree distribution (right panel). Dyadic contagion probability p=0.05. Italian population age structure. See Fig S6 for details of figure’s construction.
