# Supplementary material for: The spreading of SARS-CoV-2: Interage contacts and networks degree distribution
Source: PLoS One. 2021 Aug 25;16(8):e0256036. doi: 10.1371/journal.pone.0256036 (PMC8386875; doi:10.1371/journal.pone.0256036)
Supplement: S6 Appendix — (DOCX) [file pone.0256036.s006.docx]

# S6 Appendix. Alternative probability of dyadic contagion

| A |
| --- |
| **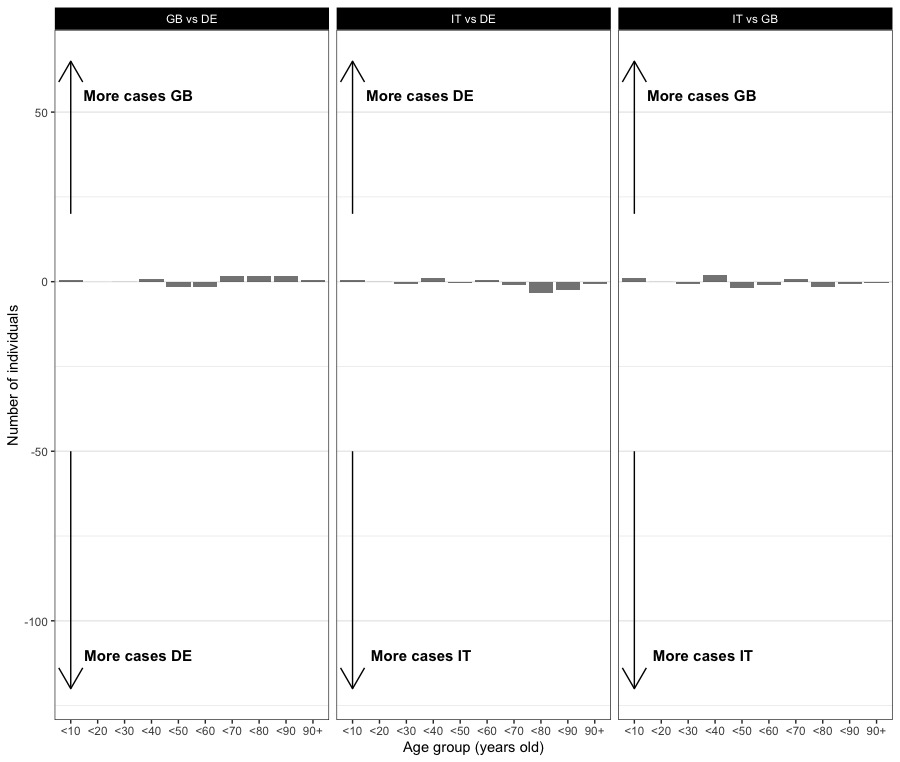** |
| B |
| **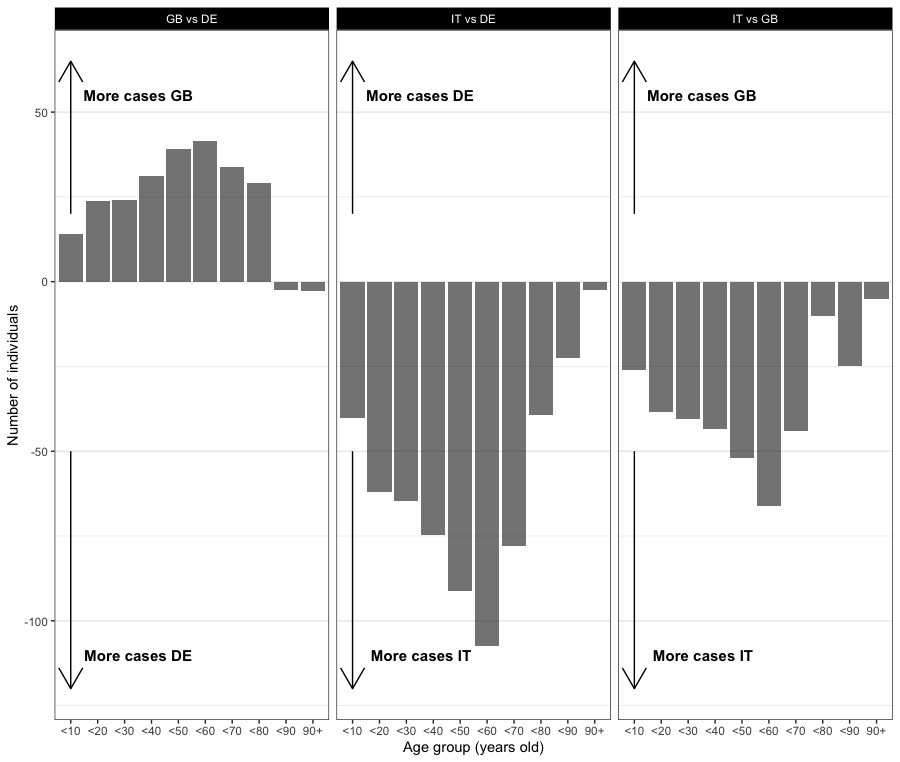** |

Figure S10: Effects of age mixing and degree distribution on diffusion with dyadic contagion probability p=0.03.

Panel (A) plots the differences attributable to age mix. Panel (B) plots the differences attributable to degree distribution. See Fig 4 and Fig 5 for details.

| A |
| --- |
| **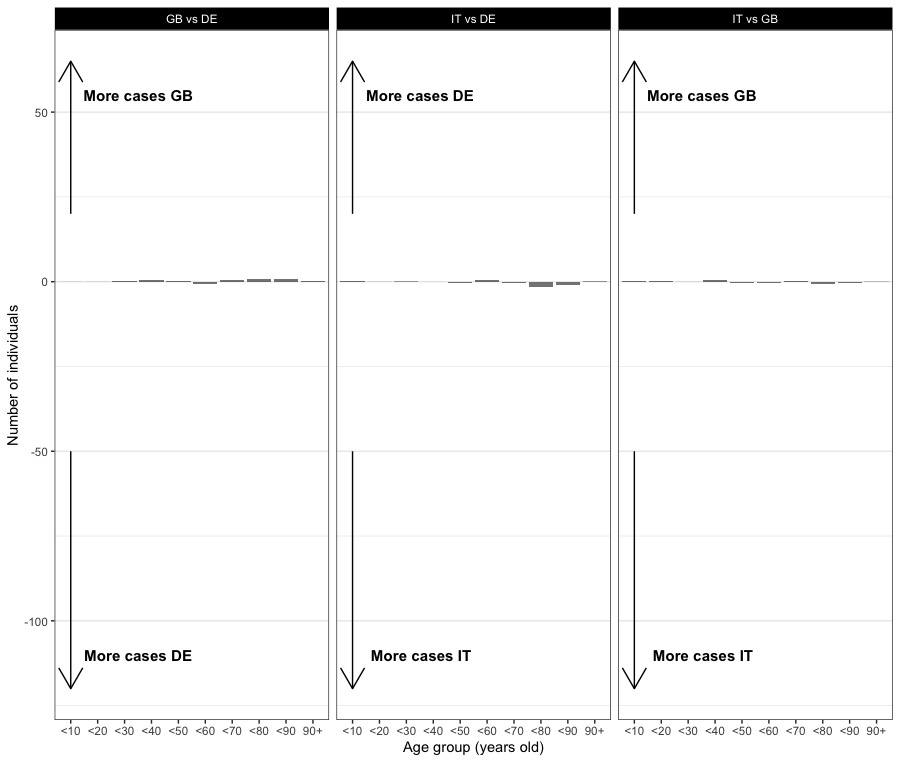** |
| B |
| **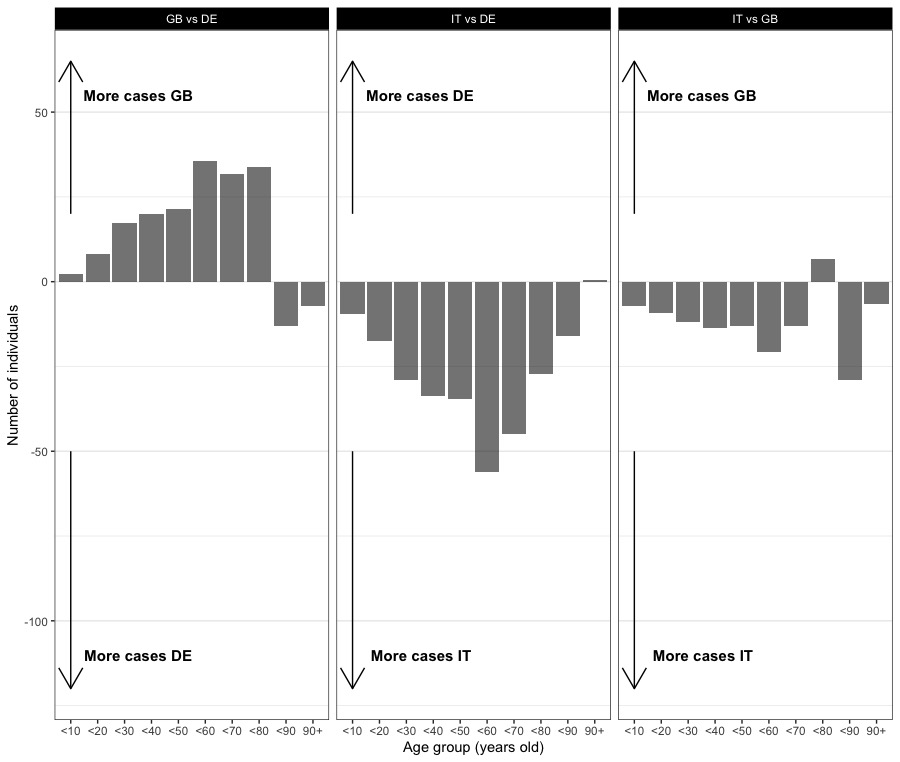** |

Figure S11: Effects of age mixing and degree distribution on diffusion with dyadic contagion probability p=0.07.

Panel (A) plots the differences attributable to age mix. Panel (B) plots the differences attributable to degree distribution. See Fig 4 and Fig 5 for details.

Not surprisingly, we find that the higher the probability of dyadic contagion, the smaller the differences between countries. Indeed, as *p* gets closer to 1 the network structure loses its importance and our simulation converges to a world in which all individuals eventually get infected. However, we observe that the qualitative result remains unchanged, and the quantitative differences rem
